# Non-contrast short MRI surveillance for HCC screening: the study protocol of the SMS-HCC prospective multicenter study

**DOI:** 10.1186/s41747-024-00432-6

**Published:** 2024-03-12

**Authors:** Céline van de Braak, François E. J. A. Willemssen, Rob A. de Man, Aad van der Lugt, Carin A. Uyl-de Groot, Daniel Bos, Roy S. Dwarkasing

**Affiliations:** 1 Department of Radiology & Nuclear Medicine, Erasmus Medical Center, Dr. Molewaterplein 40, Rotterdam, 3015GD The Netherlands; 2 Department of Hepatology, Erasmus Medical Center, Rotterdam, The Netherlands; 3 Erasmus School of Health Policy & Management and Institute for Medical Technology Assessment, Erasmus University Rotterdam, Rotterdam, The Netherlands; 4 Department of Epidemiology, Erasmus Medical Center, Rotterdam, The Netherlands

**Keywords:** Hepatitis, Hepatocellular carcinoma, Liver cirrhosis, Magnetic resonance imaging, Ultrasonography

## Abstract

Hepatocellular carcinoma (HCC) comprises 75 to 85% of all primary liver cancers. Current guidelines recommend a biannual HCC surveillance using ultrasound (US) for high-risk patients. However, due to its low sensitivity for detection of early-stage HCC lesions, there is an urgency for more sensitive surveillance tools. Here, we describe the potential of a short MRI surveillance (SMS) protocol for HCC, including axial T1-weighted in-out phase, fat-saturated T2-weighted, and diffusion-weighted sequences. In this prospective, multicenter, patient cohort study, patients will be recruited from existing HCC surveillance cohorts of six medical centers in The Netherlands. Surveillance patients who undergo biannual US, will be invited for SMS on the same day for 3 years. In case of a suspicious finding on either US or SMS, patients will be invited for a full MRI liver protocol including gadolinium-based contrast agent intravenous injection within 2 weeks. To our knowledge, this will be the first study to perform a head-to-head comparison with a paired US-MRI design. We hypothesize that the sensitivity of SMS for detection of early-stage HCC will be higher than that of US leading to improved survival of surveillance patients through timely HCC diagnosis. Furthermore, we hypothesize that the SMS-HCC protocol will prove cost-effective.

**Relevance statement** The US sensitivity for detecting early-stage HCC has been reported to be less than 50%. We expect that the proposed SMS will detect at least twice as many early-stage HCC lesions and therefore prove to be cost-effective.

**Key points**

• The low sensitivity of US necessitates better imaging tools for HCC screening.

• This is the first study with a paired US-MRI design.

• This design will allow a head-to-head comparison in both diagnostics and patient-acceptance.

• We expect that SMS can contribute to a higher survival rate.

**Graphical Abstract:**

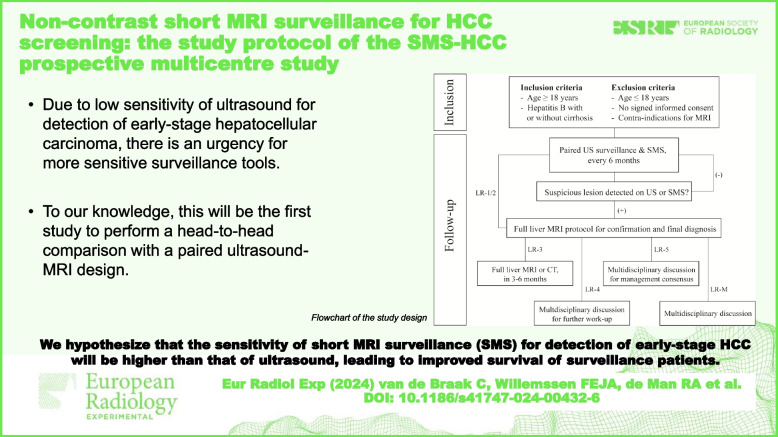

## Background

The prognosis for patients diagnosed with hepatocellular carcinoma (HCC) is poor, with a 5-year survival rate below 20% [1]. The incidence of HCC is higher among patients who have been diagnosed with chronic viral hepatitis C and/or cirrhosis [2]. The annual incidence of HCC for cirrhotic patients due to chronic hepatitis B or C infection is 2–5% [3].

According to the guidelines of the American Association for the Study of Liver Diseases−AASLD and of the European Association for Study of the Liver−EASL, biannual ultrasonography (US) is recommended for high-risk patients [4, 5]. Advantages of US as a surveillance tool include the relatively low costs, widespread availability, and no radiation exposure. However, for patients with concurrent steatohepatitis or obesity, the accuracy of US surveillance is limited, due to impaired transabdominal US access of the liver [6, 7]. In addition, a recent meta-analysis showed that the sensitivity for detecting early-stage HCC using US is merely 47% [8]. Studies and guidelines have emphasized the importance of timely detection of early-stage HCC lesions, *i.e.*, smaller than 2 cm, as this will improve survival [9–11]. Unfortunately, HCC lesions detected during US surveillance are often larger than 2 cm [8], directly emphasizing the urgent need for better surveillance methods.

The American College of Radiology states that multiphase contrast-enhanced tomography (CT) and magnetic resonance imaging (MRI) need to be considered for HCC surveillance when US is insufficient [12]. The disadvantages of both CT and MRI include high costs, long duration, and limited availability and capacity compared to US. Furthermore, the potential risks associated with the use of intravenous contrast agents, and, regarding CT, the potential risks of repeated radiation exposure should be taken into consideration.

Studies have shown that non-contrast MRI (ncMRI) has similar sensitivity for detecting focal liver lesions as compared to gadoxetic-acid enhanced MRI [13, 14]. By using ncMRI, not only the potential risks related to contrast agents are eliminated, but the costs and duration of the examination are reduced as well. These advantages may favor ncMRI as a surveillance tool.

**Fig. 1:**
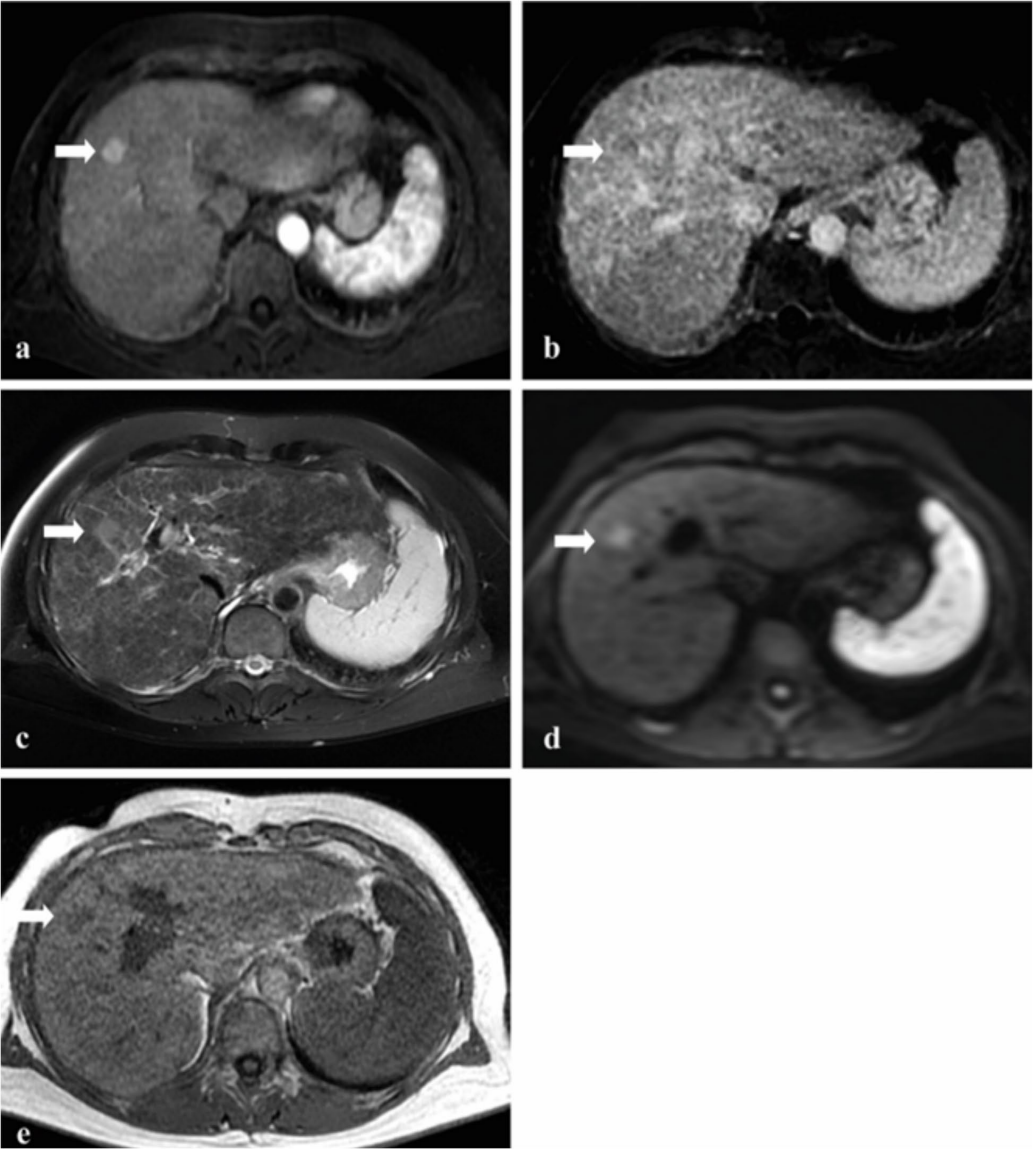
Images of a patient with chronic hepatitis C cirrhosis using the full MRI liver protocol. Newly found hypervascular lesion (arrows) measuring 13 mm in diameter can be seen (**a**), showing wash-out (**b**) and hyperintensity on T2-weighted image (**c**), as well as hyperintensity on diffusion-weighted image (**d**) and hypointensity on T1-weighted image (**e**). The lesion was classified as LI-RADS 5

Hence, we have taken the first step to investigate the feasibility of a ncMRI protocol for the screening of HCC in a simulated study, evaluating this “short MRI surveillance” (SMS) protocol in 240 patients [15]. Patients underwent yearly a full liver MRI protocol (Fig. 1), of which the three sequences constituting the SMS protocol were extracted and analyzed (Fig. 1c–e). Promising sensitivity and specificity were found of 80–97% and 72–91%, respectively. We concluded that the proposed SMS protocol is highly accurate for detecting HCC in high-risk patients.

For the next step, we will setup a prospective, multicenter study to evaluate the SMS protocol in current surveillance patients and compare it to US.

## Methods

### Study design

The study protocol was reviewed and approved by the Medical Ethics Review Committee (MEC-2022-0731). Written informed consent will be signed and obtained from all participating patients by the investigators. This trial is registered at the international trial registry of ClinicalTrials.gov (NCT05429190).

**Fig. 2:**
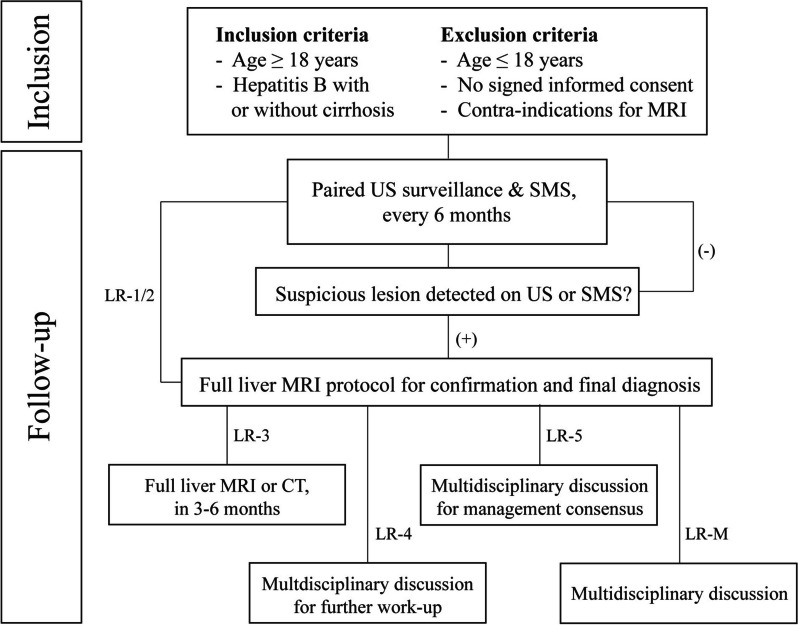
Flowchart of the study design. *HCC*, Hepatocellular carcinoma; *LR-1/5*,*M*, LI-RADS-1/5,M; *MRI*, Magnetic resonance imaging; *SMS*, Short MRI surveillance; *US*, Ultrasound

This is a prospective, multicenter, observational patient cohort study. Patients will be recruited from the already existing US surveillance cohorts of six hospitals in the Netherlands. Patients will be invited for paired US-SMS surveillance, every 6 months (Fig. 2).

### Patient recruitment and selection

The inclusion of patients will be done at (1) the Erasmus Medical Center Rotterdam; (2) the Amsterdam University Medical Center; (3) the St. Franciscus Gasthuis & Vlietland, Rotterdam; (4) the Maasstad hospital, Rotterdam; (5) the Albert Schweitzer hospital, Dordrecht; and (6) the Medisch Spectrum Twente, Enschede. The inclusion and exclusion criteria are based on the recommendations from the guidelines database from the Royal Dutch Medical Association [16] (Table 1).

**Table 1:** Inclusion and exclusion criteria

Inclusion criteria	Exclusion criteria
- Patients age ≥ 18 years at time of enrollment - Patients diagnosed with chronic hepatitis B ◦ All patients diagnosed with chronic hepatitis B and liver cirrhosis ◦ The following patients diagnosed with chronic hepatitis B without liver cirrhosis: ▪ East-Asian men ≥ 40 years of age ▪ East-Asian women ≥ 50 years of age ▪ Patients from sub-Saharan Africa ≥ 20 years of age ▪ Patients with HCC family history - Patients diagnosed with non-hepatitis B cirrhosis: ◦ Patients diagnosed with hepatitis C ◦ Patients diagnosed with alcoholic cirrhosis ◦ Patients diagnosed with hemochromatosis ◦ Patients diagnosed with primary biliary cirrhosis	- Patients age < 18 years at time of enrollment - Patients who decline to sign the written informed consent form - Patients with contraindications for undergoing magnetic resonance imaging examination

### Sample size calculation

The sample size was determined using a procedure of 1000 simulations. Based on own preliminary results and previously reported prevalence, the prevalence of HCC in our screening population was set at 12% [17–19]. Secondly, the sensitivity and specificity of the SMS protocol was assumed be 80% and 72%, respectively, based on our own data analysis [15]. For US, the sensitivity and specificity were assumed to be 53% and 91%, respectively [8, 15]. Then, the number of true and false positives and true and false negatives were generated. Using exact binomial testing, the *p*-value from comparing US and SMS was obtained. Next, this procedure was repeated by generating 1000 datasets and investigating the proportion of samples with *p*-value below 0.05 (α-level). Finally, the sample size was determined by setting the power (1-β) at 80%. This resulted in a calculated sample size of 470 assuming a drop-out rate of 10–15%.

Furthermore, given the dynamic enrollment of the participants, we estimated that the average number of screenings will be 2.5 per patients. Using the calculated sample size, the total number of screenings over the study period will be 1,175 screenings of paired US-SMS. In addition, similar prospective studies have been carried out with comparable numbers [18, 19]. Therefore, our study cohort will have a sufficient sample size to evaluate the value of the SMS and relate to other similar studies.

### Surveillance program

Patients enrolled into the study receive paired US-SMS surveillance with a 6-month interval, with an inclusion period of 3 years. The US surveillance will be performed by radiologists or hepatologists with at least 3 years of experience with US of the liver and upper abdomen on current clinical applied ultrasound systems. Participating centers have different brands of US systems with a model build 0.5 to 6 years ago. Reading of the US will be done by one reader who will also evaluate the SMS as one of three readers.

MRI will be performed on clinical applied 1.5-T system from different vendors using a dedicated body coil with an 8–16 channel range. The SMS will consist of three sequence parameters as specified in Table 2. Each SMS will be evaluated by three experienced and well-trained radiologists who will be blinded for each other’s findings and of which two readers will also be blinded for US findings.

**Table 2:** Scan parameters of the short MRI surveillance (SMS) protocol on 1.5-T systems

Sequence	Matrix size	Slice thickness (mm)	Gap (mm)	Repetition time (ms)	Echo time (ms)	Flip angle (°)	Duration (min:s)
Axial, T1-weighted 3D gradient-echo in-out phase	300 × 260	4	0	6.2	2.1/4.2	12	0:16
Axial, T2-weighted fast spin-echo with fat saturation	320 × 320	6	0.5	Dependent on triggering	93.8	160	3:42 (dependent on respiratory frequency)
Axial, echo-planar diffusion-weighted (*b* = 50, 100, and 800 s/mm^2^)	120 × 150	6	0.6	Dependent on triggering	60.1	90	2:34 (dependent of respiratory frequency)

### Imaging criteria for suspicious HCC lesions and confirmation

On US surveillance, a suspicious nodule is defined as follows:

1. A nodule larger than 1 cm, previously not seen or increased in size compared to previous US;
2. A nodule without typical features of simple cysts or hemangiomas;
3. Impression of diffuse infiltrative lesion(s) with or without portal vein obstruction;
4. Occurrence of thrombosis in previously patent portal or hepatic vein.

Reporting will be done according to LI-RADS US algorithm for visualization score and detection category of lesions.

With the SMS, a suspicious nodule is defined as a newly appearing or enlarging nodule that shows at least one of the following imaging features:

1. Hypointensity on T1-weighted imaging;
2. Hyperintensity on T2-weighted imaging;
3. Restricted diffusivity;
4. Nodule-in-nodule pattern (mosaic appearance);
5. Nodule with heterogeneous fatty changes;
6. Nodule containing blood products.

Diffuse infiltrative lesions with or without a suspected (tumor) thrombus or a new thrombus in the portal- or hepatic vein without distinct lesions will be also considered suspicious. Reporting of the SMS will be done first by quality assessment of SMS acquisition and secondly by detection category of lesions. Observations will be related to previous SMS, if available. Consensus will be reached on interpretation and registration of findings on SMS by majority vote.

In case of suspicious findings on the US or SMS, a full liver MRI protocol examination will be scheduled within 2 weeks, adopting the protocol as described by Willemssen et al. [15]. The protocol will consist of the SMS sequences and, additionally, a coronal and axial T2-weighted single-shot fast-spin echo; an axial, dynamic phase, 3D gradient echo T1-weighted image with fat suppression; contrast-enhanced axial dynamic phase, 3D gradient echo T1-weighted images with fat suppression; and a coronal, gradient echo T1-weighted image with auto calibrating reconstruction for cartesian sampling. Contrast-enhancement will be done using 7.5 cc gadobutrol 1 mmol/mL at a rate of 2 mL/sec and a saline flush of 30 mL.

In the case of undesirable full liver MRI protocol examination, then multiphase CT scan of the liver will be offered to the patient. At minimum, 64-row multidetector CT scanners from different vendors will be used. The protocol will consist of a non-contrast, late arterial, portal-venous and equilibrium phase with standard technical parameters as described by Kulkarni et al. [20]. Full MRI or multiphase CT will be evaluated and reported conform LI-RADS and discussed within the multidisciplinary liver tumor board of the respective centers for confirmation on the final diagnosis and recommendations for clinical management.

### Endpoints

The primary endpoint of this study is the sensitivity, specificity, the positive predictive value, and an estimated negative predictive value for early-stage HCC detection by SMS with respect to US surveillance during a 3-year follow-up.

A secondary endpoint is the cost-effectiveness of SMS compared to US surveillance. It is hypothesized that despite the direct higher costs of MRI, SMS will be cost-effective. We expect that SMS will detect at least twice more early-stage HCC than US, based on calculated sensitivity and specificity of the pilot study [15]. This may allow for relative less expensive treatment with favorable patient outcome when compared with specialized liver surgery and chemotherapy regimes in case of more advanced stage HCC [21]. These differences in management and inherent costs will be addressed within the calculations of the cost-effectiveness study.

Another secondary endpoint of this study is the patient acceptance of the SMS as the potential new screening standard modality. Patients will be invited to circumvent their experiences, through a questionnaire which also allows to express their compliance with both modalities.

### Data storage and management

For data storage and management, we use the national imaging platform provided by CTMM/Lygature TraIT (http://www.ctmm-trait.nl/trait-tools/xnat). This platform is built on top the open source XNAT software (www.xnat.org). Users need proper authorization in order to access images and derived data, this to secure patients data. The Clinical Trial Processor software is used to transfer data from the local PACS software to the central imaging platform. Readers of the SMS will enter their findings separately into a standardized and anonymized clinical report form in the online clinical software program Castor EDC.

### Statistical analysis

The sensitivity, specificity, positive predictive value, and an estimated negative predictive value of the SMS and US for the detection of HCC will be calculated and compared, using the results of the full MRI liver protocol as reference standard, combined with negative follow-up for the cases of no suspicious findings at US and SMS. Only an estimated negative predictive value can be calculated, due to non-availability of the full liver MRI in case of no findings on both the US and SMS. McNemar test will be used to determine significance in differences between the sensitivity and specificity of SMS *versus* US.

The cost-effectiveness analysis will be conducted from a healthcare perspective, using costs per detected cancer as outcome. Direct medical costs will be measured for both diagnostic pathways, using collected data from participating medical center databases and literature. The analysis will be carried out according to Dutch guidelines and by using a Markov model. The final outcome will be an incremental cost-effectiveness ratio to express the difference in costs between the SMS and US pathway per unit of health gain, *i.e.*, the number of detected cancer and quality adjusted life years. The uncertainty around the estimates will be addressed using deterministic and probabilistic sensitivity analyses.

In addition, data from this study and literature will be used to calculate the impact of improved detection of early-stage HCC on outcomes, *i.e.*, progression free survival and overall survival.

## Discussion

The aim of this study protocol is to evaluate the value of the SMS and compare it to the current standard of biannual US surveillance for HCC. This is a prospective multicenter study with a paired design for a single arm of patients.

Previous studies have proven the value of HCC imaging surveillance with US when compared to no screening [22, 23]. Jespen et al. [24] suggested that it may be necessary to perform more randomized controlled trials on HCC surveillance to determine whether the benefits of surveillance weigh up against the harms and the costs. A large scale randomized controlled trial showed that semiannual screening with US reduced HCC-related mortality by 37% for those who underwent surveillance, despite suboptimal adherence to the surveillance program (58.2%) [23]. Other studies have reinforced the benefits of surveillance in high-risk patients with detection of more early-stage HCC with consequently a higher rate of curative treatments and better survival than in the non-surveillance group [25, 26]. Kim et al. [27] and An et al. [28] have published study protocols based on abbreviated ncMRI protocols. Either the patients were offered an annual screening using ncMRI or were assigned to biannual US surveillance with a cross-over study design of biannual ncMRI surveillance or US [27, 28]. We believe that our study design of paired US-SMS will allow for a direct head-to-head comparison in diagnostics and patient acceptance.

To the best of our knowledge, this is the first published paired US-MRI study design. We expect that the proposed SMS will detect at least twice as many early-stage HCC lesions and be the next step in improving the hepatitis-cirrhosis HCC care chain. Based on our preliminary results and emerging insights from the literature, we expect the projected cost-effectiveness analysis to favor SMS [13–15]. We also expect the SMS to improve sensitivity for detection of early-stage HCC as an objective surveillance imaging tool, compared to US and may gain patient acceptance as a new standard for imaging surveillance of HCC.

## Data Availability

Not applicable.

## Abbreviations

CT: Computed tomography
HCC: Hepatocellular carcinoma
MRI: Magnetic resonance imaging
ncMRI: Non-contrast MRI
SMS: Short MRI surveillance
US: Ultrasound
